# Dynamics of the Sphere Model of Consciousness: Silence, Space, and Self

**DOI:** 10.3389/fpsyg.2020.548813

**Published:** 2020-09-18

**Authors:** Andrea Pintimalli, Tania Di Giuseppe, Grazia Serantoni, Joseph Glicksohn, Tal D. Ben-Soussan

**Affiliations:** ^1^Research Institute for Neuroscience, Education and Didactics, Patrizio Paoletti Foundation, Assisi, Italy; ^2^Department of History, Anthropology, Religions, Arts and Performing Arts, Faculty of Literature and Philosophy, Sapienza University of Rome, Rome, Italy; ^3^Department of Criminology, Faculty of Social Sciences, Bar-Ilan University, Ramat Gan, Israel; ^4^The Leslie and Susan Gonda (Goldschmied) Multidisciplinary Brain Research Center, Bar-Ilan University, Ramat Gan, Israel

**Keywords:** Sphere Model of Consciousness, Place of Pre-Existence, meditation, self-determination, silence

## Abstract

The *Sphere Model of Consciousness* (SMC) delineates a sphere-shaped matrix that aims to describe subjective experiences using geometric coordinates, in accordance with a neurophenomenological perspective. According to the SMC, an experience of overcoming the habitual self and the conditioning of memories could be placed at the center of the matrix, which can then be called the *Place of Pre-Existence* (PPE). The PPE is causally associated with *self-determination*. In this context, we suggest that *silence* could be considered an intentional inner environment enabling self-perception to focus on the “here and now,” which in turn improves perception of one’s own body in space. To investigate the hypotheses grounded in the model, the current preliminary study examined the Place of Pre-Existence Technique (PPEt), in which practitioners are guided to focus on a self-defined aim, reach a state of detachment from the habitual self, and envision the future. Four-hundred eighty-one volunteer PPEt practitioners completed self-report questionnaires before and after an intensive 3-day meditative training. We analyzed potential relationships between subjective experiences related to physical, emotional, mental, temporal, and spatial components, as well as self-determination and silence, before and after training. The results indicated a transition from a prevailing influence of *mental dimension* on the other aspects of experience, to a prevailing influence of the *spatial dimension*. Silence was reported more often following the training and was predominantly related to mental and emotional experiences. The results are discussed in the framework of the SMC, as compared to other models, and in relation to the shift from Narrative to Minimal Self and to increased balance among the considered dimensions.

## Introduction

**FIGURE 1:**
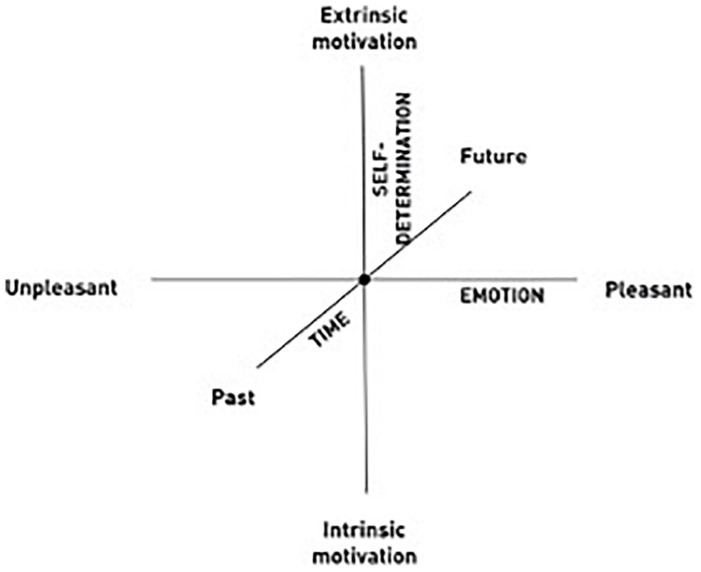
The axes of the Sphere Model of Consciousness. The *Place of Pre-Existence* (PPE) is placed at the intersection of the three axes in the center of the sphere. Adapted from Paoletti (2002a) and Paoletti and Ben-Soussan (2019).

Silence is often associated with both the Eastern and the Western meditative practices but has rarely been directly addressed in the scientific literature examining them. In the current paper, we consider the possible role of silence in the *Place of Pre-Existence Technique (PPEt)*, a meditative method based on the *Sphere Model of Consciousness* (SMC; Paoletti, 2002a, b, 2008; Paoletti and Selvaggio, 2011, 2012; Paoletti and Ben-Soussan, 2019). In the SMC, silence is conceptualized as a space or inner environment intentionally created (Ben-Soussan et al., 2019; De Fano et al., 2019) that improves perception of the ‘here and now’ (Paoletti, 2019). Similar to other models of consciousness, such as the phenomenological matrix proposed by Lutz et al. (2015) and the Default Space Model for Consciousness (Jerath et al., 2015, 2018), the SMC is a neurophenomenological geometric model considering the subjective experience of the world first of all as the experience of a body in space (Strawson, 1959, 1974). We refer here to the definition of neurophenomenology as a research program aiming to bridge the explanatory gap between first-person subjective experience and neurophysiological third-person data, through an embodied approach to the biology of consciousness (Maturana and Varela, 1980; Varela et al., 1991). In the Lutz et al. (2015) model, the geometrical matrix is specifically structured to investigate the subjective phenomenology of mindfulness practices, mapped onto a Euclidian space in which three primary dimensions are recorded: Object Orientation, Dereification, and Meta-awareness. In the Jerath et al. (2015) model, the 3D default space is composed of all cells of the body and the thalamus fills in processed sensory information from corticothalamic feedback loops, resulting in the re-creation of the internal and external worlds within the mind. The use of the sphere shape as a matrix is a distinct feature of the SMC, which refers to the geometrical properties of this figure in describing the phenomenology of subjective experience (Paoletti and Ben-Soussan, 2019) and especially the experience of non-dual consciousness (as investigated by Josipovic, 2014; Vago and Zeidan, 2016; Vieten et al., 2018). In the SMC, the three intersecting axes of a sphere spatially represent the unfolding possible dynamics and polarity of experience, in terms of temporality (past–future), emotion (pleasant–unpleasant), and self-determination, which signifies intrinsic versus extrinsic motivation (see Figure 1).

According to the model, all possible subjective experiences are placeable at the intersection of the three axes. When one intentionally distances oneself from the different aspects of one’s ongoing experience, which is placeable along the axes (e.g., worries about the future, recrimination about the past or recrimination for someone’s behavior), one achieves neutrality and detachment from the usual experience of the Minimal and Narrative Selves. As such, the new experience of equidistance from aspects of experience can be placed in the center of the sphere, which is referred to as the *Place of Pre-Existence (PPE)*. This state of neutrality and detachment from the usual experiences of the Minimal and Narrative Selves is related to a third state, realized at the center of the sphere, defined in the SMC as *Overcoming of the Self* (Paoletti and Ben-Soussan, 2019). This state is further conceptualized as a state defined in the literature as consciousness without content, or non-dual awareness (Travis and Pearson, 2000; Raffone and Srinivasan, 2009; Metzinger, 2018; Josipovic, 2019).

The concepts of Minimal Self and Narrative Self were first proposed by William James (1890/1950) and then redefined by Gallagher (2000). The former denotes the Self as “I,” the knowing subject, with a temporary presence, while the latter refers to the Self as “me,” the object that is known, which incorporates autobiographical identity related to life events and decisions (Gallagher and Zahavi, 2008 [2012]). Importantly, Berkovich-Ohana and Glicksohn (2014) further suggested that experiences related to the Narrative Self are perceived as further away from the body and more abstract and related to the future and the past. Narrative Self, Minimal Self, and Overcoming of the Self are represented in the SMC as concentric circles around the center of the sphere, with greater distance from the center signifying a more abstract experience of oneself. As the PPE is located at the center of the sphere, movement toward it dictates a shift toward the Minimal Self, or even Overcoming of the Self.

Similar to Tibetan Shamatha and Theravada Vipassana (Lindahl et al., 2014), different meditative techniques grounded in the SMC, such as exposure to *OVO Whole-Body Perceptual Deprivation* (Glicksohn et al., 2017, 2019; Ben-Soussan et al., 2019) and practice of *One Minute Meditation* (OMM, Paoletti, 2018), invite practitioners to reflect on the idea that they can reach the PPE, and thus generate positive transformation of the usual self through detachment from memories and equidistance from the dimensions of experience: spatial and temporal (Travis and Pearson, 2000; Raffone and Srinivasan, 2009; Berkovich-Ohana et al., 2013; Metzinger, 2018; Josipovic, 2019), as well as emotional, mental, and physical (Paoletti, 2008; Pesce and Ben-Soussan, 2016; Ben-Soussan et al., 2017). In addition to these dimensions, which are usually reported in relation to meditative and contemplative practices (Hinterberger et al., 2014; Wittmann, 2015; Wahbeh et al., 2018), based on the SMC, we also focused on self-determination (Paoletti and Selvaggio, 2012; Paoletti and Ben-Soussan, 2019). Intentional allocation of attention to the present moment (Wahbeh et al., 2008) is a salient component of meditation and of mindfulness practice and has also been incorporated in self-determination theory (Keune and Perczel Forintos, 2010; Ryan and Deci, 2017).

The PPEt is a guided meditation that aims to lead practitioners to a neutral perception of past and present, allowing them to envision the future from the center of the sphere, where they are assumed to be relatively free from the influence of autobiographical memories. Thus, PPEt relies on the metaphor of an *empty place* (Paoletti and Bombi, 2016) for detachment, which characterizes the state of Overcoming of the Self. This metaphor presumably induces the practitioner to overcome the dichotomous perception of emotion (pleasant versus unpleasant) and time (past versus future) (Paoletti and Ben-Soussan, 2019). In this context, intentional silence (Pinder and Harlos, 2001; Paoletti and Selvaggio, 2012; Bigo, 2018) is believed to facilitate closeness to the center of the sphere.

Subjective experience of the world is first of all a physical experience of a spatial world (Strawson, 1959, 1974). In accordance, several models of consciousness have emphasized the importance of space (Khachouf et al., 2013; Jerath et al., 2015). Based on the assumption that brain circuits related to spatial representations are involved in more complex mental constructs, and that spatial representations enable abstract thought (Rizzolatti and Sinigaglia, 2006), a correspondence between spatial coordinates and dimensions of experience is proposed in the SMC. Furthermore, spatial representation as the ground on which the mind builds abstract conceptualizations is also a central feature in self-representation according to Legrand (2006); Zaehle et al. (2007), and Blanke et al. (2015) Thus, to examine relationships between increased awareness of space and bodily self-perception in the ‘here and now’ (Legrand, 2007; Blanke et al., 2015), in the current study we examined whether intensive meditation training could affect the experience of space (Van Leeuwen et al., 2012; Hinterberger et al., 2014; Wahbeh et al., 2018) in PPEt, which would in turn alter the perception of one’s self and the level of involvement in both cognitive and emotional experience. We also examined the role of silence in the hypothesized alteration in consciousness. To this end, we utilized a self-report questionnaire in which participants were asked about changes in their experience of space and its interactions with physical, emotional, mental, and temporal experience (Metcalfe and Son, 2012; Vandekerckhove et al., 2014; Barrett, 2017; Josipovic, 2019), and open questions about spontaneous silence-related experiences between two PPEt sessions, one before and one after an intensive 3-day meditation training program.

## Method

### Participants

A total of 481 volunteers (62% women; mean age = 45.36 ± 11 years; education = 51.2% middle/high school, 48.8% undergraduate/graduate school; occupation = 49.2% self-employed and 36.5% clerical/office worker) participated in the study. All completed the SMC Meditation Practices Questionnaire (SMC-MPQ; see Section “Measures”) following two separate PPEt sessions, one before and one after a 3-day meditation course, which included theoretical instruction and nine OMM training sessions (Paoletti, 2018; see Section “Relationships Between the Constructs of the Five Dimensions and Self-Determination”). Of the 481 participants, 419 (259 women; mean age = 45.52 ± 11.36 years; education = 50.8% middle/high school, 49.2% undergraduate/graduate school; occupation = 50.1% self-employed and 34.4% clerical/office worker) attended the PPEt session on the first day of the training program, and 429 (270 women; mean age = 45.15 ± 11.29 years; education = 51.5% middle/high school, 48.5% undergraduate/graduate school; occupation = 48.3% self-employed and 38.5% clerical/office worker) attended the session on the final day of training.

The participants, who volunteered for the survey in a non-clinical, training setting, were recruited using opportunity sampling. The main aim was to carry out an exploratory study for investigating the characteristics of PPEt related to the SMC and, hence, developing new hypotheses to be explored in further studies both in clinical and empirical settings with neurophysiological tools (Smith et al., 2015). The study was approved by the Bar-Ilan University ethics committee. Participation was voluntary and required provision of written informed consent.

### Intensive 3-Day Meditation Training

Meditation training constituted an intensive 3-day course, which included three 2-h classes and OMM sessions each day. OMM (Paoletti, 2018) is a brief (1 min) meditation technique during which practitioners divide attention between breathing and envisioning the best version of themselves. Before the OMM session, several instructions are provided to prepare practitioners for meditation: (1) connect to an emotion related to self-esteem; (2) keep in mind an image associated with your best self-representation; (3) enhance an emotional state characterized by distance, detachment, and determination; (4) make a commitment to yourself; and (5) choose small actions to initiate change.

Classes addressed emotional intelligence and provided in-depth analyses of the aforementioned five points. PPEt was practiced at the beginning of the first day and again at the end of the last day of the course. Each session comprised a 20-min guided meditation, before which participants were given three instructions: (1) focus on a self-defined aim; (2) aim for deep relaxation and to reach an ‘empty space’; (3) achieve a neutral perception of the past and the present, for envisioning the future.

#### Measures

Participants were administered the SMC Meditation Practices Questionnaire (SMC-MPQ), a novel qualitative and quantitative self-report questionnaire, following each of the two PPEt sessions. The SMC-MPQ was constructed to empirically examine the SMC (Paoletti, 2002a, b, 2008; Paoletti and Ben-Soussan, 2019). It examines level of awareness of specific inner states and the ability to think critically about them, specifically in terms of the ability to analyze and evaluate evidence and arguments without bias from experience and prior knowledge (Noone et al., 2016; Holland et al., 2017).

The questionnaire focuses on the SMC constructs related to the following five dimensions:

1. Physical – The physical dimension involves physical energy, muscular state, and perceptual sensitivity (Paoletti, 2008; Paoletti and Selvaggio, 2011; Herbert and Pollatos, 2012; Farb et al., 2015);
2. Emotional – The emotional dimension refers to awareness, acceptance, and control of both positive and negative emotions, to achieve a state of balance and emotional neutrality (Gratz and Roemer, 2004; Baer et al., 2008; Paoletti and Selvaggio, 2011; Paoletti, 2013);
3. Mental – The mental dimension involves complex cognitive processes of understanding and awareness of the quality and quantity of thoughts and intuitions (Brown and Ryan, 2003; Baer et al., 2008; Paoletti, 2008; Abdoun et al., 2019);
4. Spatial – The spatial dimension refers to changes in spatial perception during practice, including awareness of the self in the surrounding space, and modification of the perception of oneself in space (Wittmann, 2013);
5. Temporal – The temporal, or time, dimension can be broken down into (1) temporal changes in time perception during practice, including awareness of the present time, awareness of elapsed time, and changes in perception of oneself over time (Wittmann, 2013; Pfeifer et al., 2016), and (2) changes in past, present, and future time perspectives, as processes that allow the practitioner to categorize and structure life events to give them coherence and meaning (Zimbardo and Boyd, 1999).

For these five dimensions, eight variables – (1) physical, (2) emotional, (3) mental, (4) spatial, (5) time perception during practice (temporal dimension), (6) present time perspective (temporal dimension), (7) past time perspective (temporal dimension), and (8) future time perspective (temporal dimension) – were each assessed by two items, one qualitative (e.g., *Briefly describe the quality and quantity of your perceptions with respect to the spatial dimension during the practice of the “Place of Pre-Existence”*) and one quantitative. More specifically, in the quantitative items, participants were asked to indicate their level of involvement on a 5-point Likert scale ranging from “not at all involved” (0) to “extremely involved” (4). Participants were also asked to think critically about how the technique had worked for them (Noone et al., 2016; Holland et al., 2017).

The SMC-MPQ also evaluates self-determination, in terms of problem-solving capacity, aspirations, and release from conditioning (Deci and Ryan, 1995; Wehmeyer, 2015). The three items in this area required participants to indicate their level of awareness on a 5-point Likert scale, ranging from “not at all aware” (0) to “extremely aware” (4).

#### Data Analysis

Preliminary descriptive and bivariate correlation analyses (Pearson correlation coefficient; two-tailed significance) were conducted, followed by a one-way analysis of variance (ANOVA; with Hochberg’s GT2 *Post Hoc* Test, Welch’s Robust Tests of Equality of Means with Games-Howell’s *Post Hoc* Test, and calculation of Holm–Bonferroni corrected *p*-values for multiple comparisons) and multiple linear regressions (MLR; stepwise, *enter* method).

Analyses were conducted for the total sample and for an age- and sex-stratified random subsample of the total sample, to enable execution of multiple comparisons. This type of sampling, which usually results in lower variance, provides a more precise estimate of main sample means and totals (Blaikie, 2003; Lohr, 2019). Ninety-two participants who attended the entire 3-day course (and completed both the ‘before’ and ‘after’ questionnaires, with no missing data) were randomly selected (evaluating margin of error, confidence level, means and standard deviations, number of strata, total population size, etc.) for inclusion in the subsample (57 women; mean age = 47.67 ± 10.47 years).

The factor structure of the SMC-MPQ was examined, as was the reliability of each variable loading on a specific factor, to enable the use of inferential statistics (Wanous and Hudy, 2001; Ginns and Barrie, 2004; Fuchs and Diamantopoulos, 2009; Diamantopoulos et al., 2012). Preliminary correlation analyses showed positive, significant (*p* ≤ 0.01) linear relationships among all eight variables. The KMO measure (>0.930) and Bartlett’s Test of Sphericity (*p* < 0.01) confirmed the adequacy of the sample. Next, the results of an exploratory factor analysis (EFA) (extraction method: principal component analysis; rotation method: promax with Kaiser normalization) revealed a two-factor model with good internal reliability [“Five Dimensions” factor (eight items): Cronbach’s *alpha* = 0.885; “Self-determination” (three items): Cronbach’s *alpha* = 0.882] and accounting for 62.2% of the total variance explained. Moreover, the two-factor model fit indices were acceptable (RMSEA = 0.064; CFI = 0.91; TLI = 0.85). Further analyses showed, for each variable, good communalities (>0.500) and high factor loading values (>0.680).

To examine the connection between silence, space (spatial dimension), body (physical dimension), and the PPEt before and after training, we first conducted a framework analysis. This qualitative data analysis (QDA), in which the texts were categorized and thematic patterns were identified (Ritchie and Lewis, 2003; Neuman, 2013; Richards, 2014), was performed with a focus on the construct of silence. As in previous research on meditative techniques, the construct of silence was operationalized based on the extent to which it, or similar terms (e.g., tranquility, vacuity, emptiness, stillness, peace, absence of noise/chaos, calmness; Cambridge Dictionary, 2019; Encyclopedia Treccani, 2019; Merriam-Webster Thesaurus, 2019), was referenced in the reports of practitioners. The concepts of “absence of thoughts and/or disturbing emotions” and “still mind” (Del Monte, 1995; Dawson, 2003; Manocha et al., 2007, 2010, 2011; Manocha, 2011; Vago and Zeidan, 2016) were also included in this context.

## Results

### Effects of Training on Constructs of the Five Dimensions and on Self-Determination

The ANOVA results indicated significant differences between the two time points (higher levels after training compared to before training) with respect to the spatial, past time perspective (temporal dimension), present time perspective (temporal dimension), future time perspective (temporal dimension), physical, emotional, and mental dimensions, as well as with respect to the self-determination variables (Table 1).

**TABLE 1:** Analysis of variance and multiple comparison results: Differences in the five dimensions and self-determination measures of the Sphere Model of Consciousness Meditation Practices Questionnaire before and after training.

Dependent variable*	*F*^1^	*df1*	*df2*	*p*	*Mean difference*	*Std. error*	*p*
Physical dimension	3.33^a^	3	1013	0.019	−0.17	0.06	0.048
Emotional dimension	6.22^b^	3	249.1	0.000	−0.25	0.06	0.000
Mental dimension	8.23^b^	3	247.2	0.000	−0.27	0.06	0.000
Spatial dimension	3.47^a^	3	955	0.016	−0.21	0.07	0.048
Past time perspective (temporal dimension)	3.51^b^	3	255.8	0.016	−0.21	0.07	0.048
Present time perspective (temporal dimension)	3.61^b^	3	236.7	0.014	−0.23	0.07	0.028
Future time perspective (temporal dimension)	8.57^a^	3	993	0.000	−0.44	0.09	0.000
Problem-solving capacity	9.80^b^	3	251.1	0.000	−0.30	0.06	0.000
Aspirations	11.11^b^	3	243.6	0.000	−0.33	0.07	0.000
Release from conditioning	12.52^b^	3	246.4	0.000	−0.39	0.07	0.000

*^1^Statistics: ^a^ANOVA; ^b^Welch’s Robust Tests of Equality of Means. ^2^Multiple comparisons: ^c^Hochberg’s GT2 Post Hoc Test with equal population variances; ^d^Games-Howell’s Post Hoc Test with unequal population variances. ^∗^Only significant differences reported; p < 0.05. Holm–Bonferroni adjusted p-values were calculated for all comparisons.*

### Relationships Between the Constructs of the Five Dimensions and Self-Determination

The preliminary bivariate correlation analysis showed positive, significant (*p* ≤ 0.01) linear relationships among all eight variables related to the SMC constructs.

**FIGURE 2:**
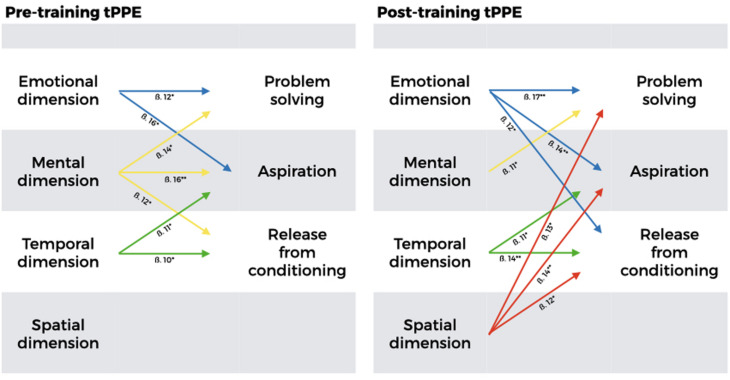
Graphic representation of multiple linear regression models (dependent variables: self-determination; independent variables: emotional, mental, spatial, and temporal dimensions). PPEt, technique of the Place of Pre-Existence. *p ≤ 0.05, **p ≤ 0.01. See Tables 2A,B for further details. Note that while the temporal dimension remained the same, the spatial dimension only appeared after training.

Further investigations with MLR revealed the following significant relationships among participants who attended the pre-training PPEt session (see Table 2A and Figure 2):

**TABLE 2:** Multiple linear regression models with Sphere Model of Consciousness Meditation Practices Questionnaire self-determination measures as dependent variables.

*Model*	*Independent variable*	β	*t*	p*
**(A) Pre-training (*n* = 419)**				
Model 1: Problem-solving capacity as dependent variable^a^				
	Emotional dimension	0.12	2.10	≤0.05
	Mental dimension	0.14	2.44	≤0.05
Model 2: Aspirations as dependent variable^b^				
	Emotional dimension	0.16	2.00	≤0.05
	Mental dimension	0.16	2.77	≤0.01
	Time perception (temporal dimension)	0.11	2.10	≤0.05
Model 3: Release from conditioning as dependent variable^c^				
	Mental dimension	0.12	2.11	≤0.05
	Time perception (temporal dimension)	0.10	1.97	≤0.05
**(B) Post-training (*n* = 429)**				
Model 4: Problem-solving capacity as dependent variable^a^				
	Emotional dimension	0.17	3.17	≤0.01
	Mental dimension	0.11	2.26	≤0.05
	Spatial dimension	0.13	2.47	≤0.05
Model 5: Aspirations as dependent variable^b^				
	Emotional dimension	0.14	2.67	≤0.01
	Spatial dimension	0.14	2.59	≤0.01
	Time perception (temporal dimension)	0.11	2.02	≤0.05
Model 6: Release from conditioning as dependent variable^c^				
	Emotional dimension	0.12	2.22	≤0.05
	Spatial dimension	0.12	2.13	≤0.05
	Time perception (temporal dimension)	0.14	2.77	≤0.01

Model 1^a^ = adjusted R^2^ = 0.08, F(5,393) = 7.06, p ≤ 0.01; Model 2^b^ = adjusted R^2^ = 0.09, F(5,392) = 8.07, p ≤ 0.01; Model 3^c^ = adjusted R^2^ = 0.07, F(5,390) = 6.23, p ≤ 0.01; Model 4^d^ = adjusted R^2^ = 0.11, F(5,404) = 10.33, p ≤ 0.01; Model 5^e^ = adjusted R^2^ = 0.10, F(5,402) = 8.68, p ≤ 0.01; Model 6^f^ = adjusted R^2^ = 0.10, F(5,403) = 8.70, p ≤ 0.01. *Only significant results are reported; p < 0.05. All reported correlations were positive; p ≤ 0.01.

• Emotional dimension (IV) and problem-solving capacity and aspirations (DV).
• Mental dimension (IV) and problem-solving capacity, aspirations, and release from conditioning (DV).
• Time perception (Temporal dimension) (IV) and aspirations and release from conditioning (DV).

Furthermore, the preliminary bivariate correlation analysis, among participants who attended the post-training PPEt session, showed positive, highly significant (*p* ≤ 0.01) linear relationships among all eight variables related to the SMC.

The following significant relationships were found after performing MLR analysis (Table 2B and Figure 2):

• Emotional dimension (IV) and problem-solving capacity, aspirations, and release from conditioning (DV).
• Mental dimension (IV) and problem-solving capacity (DV).
• Spatial dimension (IV) and problem-solving capacity, aspirations, and release from conditioning (DV).
• Time perception (Temporal dimension) (IV) and aspirations and release from conditioning (DV).

Based on theoretical hypotheses regarding how PPEt works, the aim of the MLR analyses was to evaluate possible relationships of the variables related to the SMC constructs with changes in the personal level of awareness of a personality characteristic, such as self-determination ability. Table 3 shows the personal reports of the 429 participants, who participated in the PPEt session on the last day of training, to better understand the quality of the variables related to the SMC constructs associated with the five dimensions.

**TABLE 3:** Personal reports of the 429 participants who attended the PPEt session on the final day of training.

*Category*	*Self-reports*
*Physical dimension*	*“I perceived my energy state more clearly; the muscles relaxed; I clearly felt the limits of the body perimeter.”* (#384) *“I felt a very deep relaxation; I felt expansion.”* (#393)
*Emotional dimension*	*“I felt how much love and how many mistakes I had made in life and I wanted to dissolve them. I was moved as I went back to the place of pre-existence where I was peaceful, instead.”* (#147) *“Moments of mental silence, surprise at the possibility of transforming some negative/stressful emotions to neutral.”* (#149)
*Mental dimension*	*“My thoughts were constructive and solution-oriented, then they became silence and listening.”* (#160). *“The mind had no thoughts; it was there, it didn’t worry, it didn’t speak, it was listening and enjoying the here and now.”* (#255)
*Spatial dimension*	*“I perceived space more as if there were no obstacles, as if we were one.”* (#6) *“It seemed to me that I was no longer limited by the body, but part of the whole.”* (#236)
*Time perception (temporal dimension)*	*“Time expanded; it was no longer as before; everything was wrapped in light and gratitude; I felt powerful and capable.”* (#252) *“I felt that time had no continuity, it was a single flow, the various moments seemed consequential.”* (#282)
*Past time perspective (temporal dimension)*	*“I saw myself in the past: I did not judge the events of my life. Events had lost their sign and therefore their intensity.”* (#65)*“I felt true happiness in remembering positive events and neutrality in remembering negative events as they had no longer strength and influence on me.”* (#394)
*Present time perspective (temporal dimension)*	*“I felt the body, the breath, the flow of energy.”* (#204) *“The present as the transition, the point of contact between the past and the future. Being able to flow through the past to support the present and build the future. Live, not theoretical, experience during practice.”* (#10)
*Future time perspective (temporal dimension)*	*“I felt expanded space, trust, hope, quiet, promise.”* (#256) *“Feeling of having dissolved my conditionings and being able to live more freely and fully, without constrictive confrontations; full power of myself.”* (#282)

*Analyzed categories: physical, emotional, mental, spatial, temporal, past present and future time perspectives dimensions. #Participant number.*

### Silence and Related Variables

The QDA focusing on the construct of silence investigated potential associations between this state and the other variables. Following the pre-training PPEt session (*n* = 419), 58 participants made reference to silence, in relation to the following variables: (1) mental state (37/58; e.g., “My thoughts were free from judgments, memories, and mental schemes; the noise disappeared, and silence appeared,”); (2) emotional state (12/58; e.g., “I experienced the redefinition and focalization of the emotions, bringing them to a neutral and useful condition,” “I felt the transition from positive to neutral emotions as breathing became calmer and inner peace increased,” “I felt positive emotions of peace, possibility, trust and compassion.”); (3) critical thinking about the technique’s effects (9/58; e.g., “The technique took me to a neutral place without time or space, where I find the possibility of starting a new story.”). Of the participants who attended the post-training PPEt session (*n* = 429), 115 referred to silence, in relation to the following variables: mental state (51/115), emotional state (38/115), and critical thinking regarding the ways the technique worked for them (26/115).

## Discussion

### Shifting From the Mental to the Spatial Dimension

According to Strawson (1959, 1974), our subjective experience of the world is, in the first place, a physical experience of a spatial world, and the primary process in self-consciousness is convergence of perception and action into one’s body and its location in space (Blanke and Metzinger, 2009; Limanowski and Blankenburg, 2013). In this context, it is noteworthy that expanded embodiment and sense of presence are at least partially reflected in physical balance and spatial orientation; more integrative transformations of consciousness associated with meditative and lucid dreaming are correlated with superior performance, while the opposite is true for disintegrative states (Hunt, 2007).

These assumptions led to the concept of embodied cognition, of which Varela et al. (1991) are among the foremost proponents. According to this approach, the body is both physical and subjective, such that one’s body does not become an object like any other perceived object. Although the body becomes objective by being perceived, the perceivability of the body makes it subjective (Menon, 2016). Moreover, in the model proposed by Jerath et al. (2018), the formation of a 3D body space within the mind is considered the very basis of consciousness. Khachouf et al. (2013) pointed out that pre-reflective self-awareness includes a sheer bodily component with affective valence, associated with the insula and anterior cingulate cortex, which are often activated in concert, and which have been viewed as limbic analogs of the sensory and motor cortices, respectively (Heimer and Hoesen, 2006; Craig, 2009). This interpretation fits nicely with the role of these regions in providing a bodily component of sense of self, related to homeostatic and basic sensorimotor loops. Within the search for neural structures underlying embodied transcendental features of consciousness, Khachouf et al. (2013) also note that it would not be exhaustive to consider the historical/autobiographical sense of self as the ultimate *a priori* structure to which extrinsic stimuli should conform in order to become conscious. In this context, it has further been observed that Narrative Self is phenomenologically placed further away from the body, in the domain of abstract thought (Edelman and Tononi, 2000; Berkovich-Ohana and Glicksohn, 2014).

While some have found that increased consciousness is accompanied by increased spatial perception (Wahbeh et al., 2008; Hinterberger et al., 2014; Blanke et al., 2015), others have found it to be related to decreased spatial boundaries (Baars, 1998; Travis and Pearson, 2000; Metzinger, 2018). Consequently, the main aim of the current study was to investigate the change in the experience of spatial, temporal, emotional, mental, and bodily perception, as well as the experience of silence and self-determination through self-reporting, in light of the SMC. The main finding was that before training, PPEt experiences related to the spatial dimension did not appear to influence other dimensions, while after training, the spatial dimension influenced the self-determination variables, especially, problem-solving capacity, aspirations, and release from conditioning (see Figure 1). This result suggests a shift from the Narrative Self, in which the mental dimension is more influential, to the Minimal Self, in which the spatial dimension is predominant. This shift in the phenomenology of consciousness supports the influence of the spatial dimension on other dimensions after training.

Experience of the Minimal Self as interoceptive (Legrand, 2006; Blanke and Metzinger, 2009; Limanowski and Blankenburg, 2013) seems to be strengthened at the expense of the Narrative Self through meditative practice (Dolan, 2004; Farb et al., 2007; Ben-Soussan et al., 2015). The SMC, on which the meditative techniques utilized in this study are based, refers to a distinction between primary sensory (and thus spatial) and higher-order sensory consciousness (Edelman and Tononi, 2000), related to Minimal and Narrative Selves, respectively. More specifically, the proposed correspondence between spatial coordinates and dimensions of experience is based on the assumption that sensorimotor circuits underlie higher-order cognitive processes (Rizzolatti and Sinigaglia, 2006). The current results are, therefore, consistent with the SMC, which delineates spatial dimensions providing a visual matrix in which mental contents can be placed. This should also be true with regard to the effect of training on the spatial dimension, as the perception of oneself in space would represent the neurophenomenological substrate of Minimal Self-awareness (Varela et al., 1991; Legrand, 2006; Blanke and Metzinger, 2009; Khachouf et al., 2013; Limanowski and Blankenburg, 2013; Jerath et al., 2018).

### Silence and a Possible Shift Toward the Minimal Self

The second aim of the study was to examine experiences related to silence, and their relationship to the other dimensions studied. The results demonstrated that participants made more references to silence in their post-training reports (14% to 27%). Silence-related reports regarding the mental dimension decreased in favor of both emotional and critical thinking about the practices. This suggests that intentionally sought silence indeed has a role in the shift toward the Minimal Self, as represented in the SMC.

In this regard, silence could have a significant role in reducing the self-confirmatory loop of the self, as noted by Khachouf et al. (2013). They point out that a shared characteristic of many contemplative practices is the instruction to maintain steady posture and minimize the variability of sensory input, for example by maintaining gaze direction and choosing a silent environment. Cognitive activity related to external input, which is generated through adjustment of an internal probabilistic model based on sensory data, becomes cognitive activity about the self (Khachouf et al., 2013), which could be conceptualized as a shift of attention from the external to the internal environment (Ben-Soussan et al., 2019; De Fano et al., 2019; Paoletti and Ben-Soussan, 2020, in press). It has further been suggested that by intentionally entering an (inner and outer) state of silence, we gradually move toward the center of the sphere (Paoletti and Ben-Soussan, 2019; Paoletti et al., 2020), which is also characterized by timelessness and spacelessness (Vaitl et al., 2005; Studerus et al., 2011; Wittmann, 2015). Consequently, the current study and subsequent examinations can have practical implications in the context of cognitive psychotherapies. Future investigations are required to examine whether additional dimensions are involved in achieving greater silence, and whether silence is specifically related to greater equilibrium among the physical, emotional, and mental dimensions. Additional work might also aim to increase our understanding of the different aspects of silence, including its possible use as a suggestion, psychological effects, and their physiological correlates.

### Concluding Remarks and Limitations

The current preliminary study was the first study to examine the PPEt. A clear limitation involves our inability to distinguish between the effects of the OMM and PPEt practices and the lack of another meditation-based intervention and a waitlist control group. Thus, the increased influence of spatial dimension, assumed to be a result of PPEt, could be related to OMM as well, or to expectation bias. Future work with additional study and control groups, in which the current and additional practices are tested separately, can potentially isolate the specific effects of each.

Additional studies should further investigate the neurophysiological correlates of both techniques, and their possible synergistic effects. Furthermore, while open self-report methods are important, it might be beneficial to combine them with additional structured questionnaires, such as the Inventory on Subjective Time, Self, Space (STSS; Jokic et al., 2018) as well as electrophysiological measures. We are currently working in these directions. Finally, while it is difficult to examine the possible differential effects of training on various psychological profiles and their electrophysiological correlates, in group settings, additional studies could examine this in the lab.

## Data Availability Statement

All datasets presented in this study are included in the article/supplementary material.

## Ethics Statement

The studies involving human participants were reviewed and approved by Bar-Ilan University Ethics Committee. The participants provided their written informed consent to participate in this study.

## Author Contributions

AP, TD, TB-S, and JG contributed to the conception and design of the study. GS organized the database and performed the statistical analysis. AP, TD, and TB-S wrote the manuscript. JG and GS contributed to the manuscript revision and read and approved the submitted version. All authors contributed to the article and approved the submitted version.

## Conflict of Interest

The authors declare that the research was conducted in the absence of any commercial or financial relationships that could be construed as a potential conflict of interest. The handling editor declared a shared affiliation, though no other collaboration, with one of the authors AP the at time of review.
